# Psychedelics and Autism Therapy: A Review of Current Research and Future Directions

**DOI:** 10.3390/cimb48040417

**Published:** 2026-04-18

**Authors:** Christopher S. Gondi, Manu Gnanamony, Tarun P. Gondi, Lilyt Nersesyan, Lusine Demirkhanyan

**Affiliations:** 1 Departments of Internal Medicine, University of Illinois College of Medicine Peoria, Peoria, IL 61605, USA; 2 Departments of Surgery, University of Illinois College of Medicine Peoria, Peoria, IL 61605, USA; 3 Departments of Health Science Education and Pathology, University of Illinois College of Medicine Peoria, Peoria, IL 61605, USA; 4 Health Care Engineering Systems Center, The Grainger College of Engineering, University of Illinois at Urbana-Champaign, Urbana, IL 61801, USA; 5 Departments of Pediatrics, University of Illinois College of Medicine Peoria, Peoria, IL 61605, USA; 6 School of Communication and Media, University of Illinois Springfield, Springfield, IL 62703, USA; 7 Department of Clinical Psychology, College of Professional Psychology, The Chicago School, Chicago, IL 60654, USA

**Keywords:** Autism Spectrum Disorder (ASD), psychedelics, neuroplasticity, serotonin signaling, therapeutic potential

## Abstract

Autism Spectrum Disorder (ASD) is a lifelong condition marked by challenges in social communication and repetitive behaviors. Current treatments, primarily behavioral therapies, often fail to address the core symptoms. Recent research has explored the potential of psychedelics, such as LSD, psilocybin, and MDMA, as a new therapeutic approach. While these substances primarily modulate the serotonin 5-HT_2A_ receptor, their therapeutic effects also involve interactions with other serotonergic, dopaminergic, and glutamatergic pathways, collectively promoting neuroplasticity—the brain’s ability to change and adapt. The specific receptors’ activation leads to structural and functional changes in the brain that can enhance social behavior and emotional regulation. Studies show that psychedelics may reduce symptoms of conditions like treatment-resistant depression and PTSD, highlighting their therapeutic potential. For ASD specifically, psychedelics may improve psychological flexibility, reduce distress, and enhance social interaction. While promising, the use of these substances requires careful consideration. Psychedelics can induce intense experiences and altered states of consciousness, necessitating strict monitoring and support during therapy. Ethical guidelines, including informed consent, are crucial, especially for vulnerable populations. In conclusion, psychedelics hold significant promise for treating ASD and other psychiatric disorders by promoting neuroplasticity and modulating complex signaling pathways. Continued research and clinical trials, conducted with strong ethical oversight, are essential to realizing their full therapeutic potential.

## 1. Introduction

Autism Spectrum Disorder (ASD) is a lifelong neurodevelopmental condition characterized by difficulties in social communication and interaction, alongside restricted, repetitive behaviors [1,2]. In clinical practice, there are no medications specifically designed to treat the core symptoms of ASD; management primarily involves behavioral therapies, such as applied behavioral analysis and occupational therapy [3,4]. These interventions are delivered by multidisciplinary teams including psychologists, psychiatrists, and therapists [5,6]. However, many patients continue to struggle with social challenges even after years of behavioral therapy. While current pharmacological interventions—such as those for co-occurring depression or anxiety—alleviate specific symptoms, they fail to address the underlying social and communication difficulties of ASD [7,8,9,10,11].

The limitations of current strategies have led to increasing interest in the potential of psychedelics—serotonergic hallucinogens that produce mind-altering effects such as hallucinations and intense emotional experiences [12]. Notable examples include lysergic acid diethylamide (LSD) [12], psilocybin [12,13,14], dimethyltryptamine (DMT) [12,15,16,17,18,19], and 3,4-methylenedioxymethamphetamine (MDMA) [20,21]. Recent evidence suggests that psychedelic-assisted therapy, which combines moderate dosing with professional psychological support, could be a breakthrough for various mental health conditions [14,22,23,24,25,26,27,28,29,30,31,32,33,34,35,36,37]. For instance, clinical trials have shown that psilocybin can produce rapid and sustained antidepressant effects in treatment-resistant patients [15,38,39,40,41,42]. Given that the core symptoms of ASD—such as repetitive thoughts and social anxiety—share underlying neurobiological abnormalities with these conditions, the scientific community has begun exploring psychedelics as a novel therapeutic avenue for the autistic brain [43,44,45,46,47,48,49,50,51].

Unlike many conventional drugs, psychedelics act primarily as agonists at the serotonin receptors, particularly the 5-HT_2A_ subtype, to alter cognition and perception [16,52,53]. This modulation is thought to promote neuroplasticity, the brain’s innate capacity to reorganize and adapt [54]. The primary purpose of this review is to evaluate the clinical potential of serotonin agonists and other psychedelic substances for managing the core symptoms of ASD. By exploring properties such as neurogenesis and the regulation of monoamine neurotransmitters like serotonin and dopamine, this review elucidates the biological foundation for using these substances to improve psychological flexibility and social interaction [55,56]. Furthermore, this manuscript synthesizes emerging evidence from preclinical and human studies while addressing the critical legal and ethical considerations necessary for future clinical applications.

## 2. Some Known Psychedelics

Psychedelics, a class of psychoactive substances, have long captivated human curiosity and exploration [57]. These compounds induce profound alterations in perception, cognition, and consciousness, often accompanied by vivid sensory experiences and heightened introspection. Among the well-known psychedelics are lysergic acid diethylamide (LSD), renowned for its potent hallucinogenic effects [44,47]; psilocybin, found in certain species of mushrooms and revered for its introspective and mystical qualities [14,28,38,39,40]; and dimethyltryptamine (DMT), known for inducing intense, short-lived visionary experiences (Table 1). Other notable psychedelics include MDMA, mescaline, and ketamine, each with unique psychoactive profiles and potential therapeutic applications [18,19,58]. Despite their diverse origins and effects, psychedelics share a common thread of altering perception and fostering profound shifts in consciousness, making them subjects of ongoing scientific inquiry, cultural fascination, and therapeutic exploration.

### 2.1. LSD

Lysergic acid diethylamide, commonly known as acid, is a potent psychedelic substance. LSD was first synthesized in 1938 by the Swiss chemist Albert Hofmann at the Sandoz Laboratories in Basel, Switzerland. It is renowned for its ability to induce profound alterations in perception, mood, and consciousness. LSD, typically consumed orally, can include visual hallucinations, intensify sensory experiences, and alter thought patterns [13,16,53]. The subjective experience of LSD often involves a heightened sense of interconnectedness and ego dissolution, leading to profound introspection and mystical experiences. Despite its recreational use, LSD has also been investigated for its potential therapeutic benefits, particularly in the treatment of psychiatric disorders such as depression, anxiety, and PTSD [33,59]. However, its use also carries risks, including the potential for adverse psychological reactions and triggering latent mental health conditions. As such, LSD remains a subject of both scientific research and cultural fascination. The potential therapeutic benefits of LSD in society, derived from empirical evidence in research studies, remain enigmatic. There is a growing public interest in psychedelic research, with numerous studies highlighting positive mental health outcomes and personal experiences being showcased in mainstream media. However, due to strict regulatory constraints, only a limited number of scientifically rigorous clinical trials adhering to safety and ethical guidelines have been published thus far [60]. These trials have primarily focused on challenging patient populations, yielding promising results. The existing evidence, derived from both preliminary and randomized, double-blind trials, indicates that LSD could revolutionize the treatment of depression and addiction [59]. The use of lysergic acid diethylamide in the management of autism is a highly intriguing and promising area of exploration that calls for extensive research. However, it is imperative to acknowledge that very limited scientific studies have been undertaken or legally ventured into to comprehensively understand and address the potential value of LSD in autism management. Because of this, there remains a substantial need for further research endeavors to ascertain the true efficacy and benefits of LSD as a potential therapeutic tool for individuals on the autism spectrum.

### 2.2. Psilocybin

Psilocybin [12,13,14,40,42] acts as a potent agonist at a specific serotonergic receptor site called the 5-HT_2A_ receptor. This receptor plays a vital role in regulating sensory input, cognitive processes such as working memory, and actions and decision-making [61,62,63,64]. By stimulating activity at these receptor sites, psilocybin can alter the brain’s processing of sensory information and the perception of the self and the external environment. This phenomenon, known as neural plasticity, refers to the nervous system’s ability to reorganize its structure, function, and connections in response to factors such as environmental changes, development, or injury. In individuals with autism spectrum disorder, psilocybin has shown promise in promoting neural plasticity, resulting in improvements in cognitive functioning and social interaction skills. Studies in animals have suggested that psilocybin and related compounds could potentially reduce harmful brain inflammation. This reduction is believed to occur by activating specific receptors, the 5-HT_2A/C_ receptors, which belong to the brain’s serotonergic receptor family (Table 2). By reducing microglial activation, the 5-HT_2A/C_ receptors trigger an anti-inflammatory response from the brain’s immune system and facilitate repair of any existing inflammation-related damage [65,66,67,68,69,70,71,72,73]. This anti-inflammatory response may contribute to the potential therapeutic effects of psilocybin in managing symptoms of ASD. Future research may explore the potential use of psilocybin in therapeutic approaches aimed at reversing inflammation associated with ASD during early life. This is an important step in enhancing the prevention or reduction of ASD symptoms, as the developing brain has a heightened ability to self-repair and regenerate. However, it is important to note that current research on the clinical use of psilocybin, including its management of ASD, is limited. Any unauthorized use of this drug outside approved research studies may constitute a criminal offense.

### 2.3. DMT

Dimethyltryptamine [12,16,18,57] is a naturally occurring psychedelic substance that shares a similar chemical structure with serotonin. DMT is found in ‘ayahuasca’, a traditional shamanic brew used in the Amazonian region for many years as a means of healing the mind and body [13,60,69]. Recently, ayahuasca has become popular in the Western world, leading to the rise of “ayahuasca retreats” [74]. These retreats aim to create a safe and controlled environment for individuals to explore the potential therapeutic benefits of DMT for various mental health conditions, including ASD [12]. Participants often report profound insights, heightened self-awareness, and a stronger sense of connection with themselves and others. In the United Kingdom, DMT is classified as a Class A drug under the Misuse of Drugs Act 1971, which indicates that it is considered highly harmful and addictive. Possessing, supplying, or producing DMT illegally can result in severe penalties. In countries like the United States, DMT is classified as a Schedule I drug, signifying a high potential for abuse and no accepted medical use (Drug Scheduling-Drug Enforcement Agency). Despite its controversial legal status, research on the therapeutic effects of DMT has been increasing. Evidence suggests that DMT may have fast-acting and long-lasting antidepressant properties. A recent study published by Muttoni and colleagues in 2019 found that a single dose of ayahuasca significantly reduced depression scores in individuals with recurrent depression [75]. These findings offer promise for future research, as they can help predict treatment responses in mood and depressive disorders. Compared to conventional antidepressants that often take weeks to months to take effect, the quick onset and enduring impact of DMT- and ayahuasca-assisted therapy suggest that DMT may revolutionize depression treatment. However, the exact mechanisms by which DMT produces its rapid and sustained antidepressant effects are not completely understood. Current evidence indicates that DMT interacts with serotonin receptors, particularly the 5-hydroxytryptamine (5-HT) receptor subtype family [76,77,78,79]. By directly stimulating these receptors, DMT can enhance serotonin neurotransmission, leading to increased neuron activity in regions of the brain responsible for mood and emotions. This mechanism is similar to how many modern antidepressants work. DMT is also believed to act as a potent agonist of the sigma-1 receptor (Table 2), a protein involved in various brain functions, including calcium transport and cellular survival pathways. By stimulating this receptor, DMT may activate neuroprotective and anti-inflammatory processes in the brain, promoting neural plasticity and resilience against stress and mood disturbances. However, more research is needed to confirm these proposed effects of DMT on the sigma-1 receptor and its relevance to depression treatment. Further studies should also explore whether DMT has the potential to alleviate the core symptoms of ASD and improve social communication and interaction skills.

### 2.4. MDMA

MDMA, known as 3,4-methylenedioxymethamphetamine, is an illegal substance widely used for recreational purposes in Western society due to its potent psychoactive effects. However, there has recently been a surge in interest and research into the potential therapeutic benefits of MDMA for various mental health conditions, specifically Post-Traumatic Stress Disorder [21,32,34,36,37,80,81] and ASD [56,82,83,84]. MDMA is a synthetic drug that acts as a strong monoamine releaser, enhancing the release of neurotransmitters like serotonin, dopamine, and norepinephrine in the brain. While the exact mechanism of action is not fully understood, it is believed that MDMA primarily affects the serotonergic system, which plays a critical role in mood regulation, anxiety, and social behaviors [85]. Because of this, MDMA is an intriguing candidate for pharmacological interventions in mental health disorders such as ASD [56,83], as serotonin is crucial for neural plasticity and normal brain development. Animal studies have shown that MDMA can effectively reduce repetitive, self-injurious, and stereotypical behaviors while improving social interactions and reducing anxiety. Furthermore, there is evidence suggesting that MDMA increases the release of oxytocin, also known as the “trust hormone,” which is essential for social bonding, regulating social behaviors, and managing anxiety (Table 2). Interestingly, comprehensive studies on neurotransmitters have revealed that MDMA triggers a temporary yet potent release of both oxytocin and vasopressin, another hormone linked to complex social and affiliative behaviors. This surge of oxytocin can promote trust and alleviate the negative effects of social interactions, which is particularly relevant for individuals with ASD who may experience heightened social anxiety and fear. Additionally, research has shown that administering MDMA to individuals with ASD can improve emotional recognition, empathy, and social cognition, providing further evidence of its potential as a therapeutic option for managing the symptoms of ASD [56]. One randomized controlled trial conducted by Danforth et al. found that MDMA-assisted therapy resulted in significant reductions in social anxiety and interpersonal difficulties among adults with autism [82,86].

### 2.5. Ibogaine

Ibogaine has been extensively studied as a potential treatment for ASD because of its unique pharmacological composition [13,87,88]. It is a psychoactive substance derived from the root bark of the African *Tabernanthe iboga* plant, traditionally used in West-Central Africa for initiation ceremonies and spiritual rituals [89,90,91,92,93,94,95]. The compound is classified as a Schedule I controlled substance in the United States, making it illegal to manufacture, distribute, dispense, or possess. The legal status of ibogaine varies globally, with certain European countries like the Netherlands, along with Brazil, Mexico, and Canada, having less regulation [96]. Some private clinics providing ibogaine therapy for opioid dependence and other substance use disorders are located in Mexico and Canada, where regulations permit such practices. Nevertheless, it is crucial to acknowledge that these treatments lack FDA approval and have been associated with serious adverse events and even fatalities [97,98,99]. Despite these risks, the distinct characteristic of ibogaine lies in its impact on various neurotransmitter systems, including serotonin, dopamine, and glutamate. Its notable capability to reduce drug cravings and withdrawal symptoms is primarily attributed to its influence on opioid receptors and modulation of neuroplasticity [30,100,101,102,103,104,105,106,107]. Furthermore, research indicates that ibogaine not only has antagonistic effects but also agonistic interactions with nicotinic acetylcholine receptors [108,109,110,111,112,113,114]. As abnormalities in cholinergic transmission have been linked to cognitive and emotional impairments in ASD [115,116,117,118], the modulation of these receptors by ibogaine could be particularly beneficial in managing specific aspects such as cognitive inflexibility and impulsivity. Additionally, recent findings indicate that the metabolite of ibogaine, noribogaine [119,120], acts as an allosteric modulator of sigma-2 receptors [119]. This mechanism could potentially stabilize mood and anxiety dysregulation associated with ASD since sigma-2 receptor selective ligands have demonstrated effectiveness in mood and anxiety-related disorder therapy [120,121,122,123]. However, caution is necessary, as translating these findings from preclinical studies to clinical practice for ASD is intricate and uncertain. Promising results from animal studies and non-controlled, open-label trials have shown safety, but extensive, well-controlled research is required to comprehensively examine treatment duration and long-term outcomes before determining the true benefits of ibogaine for individuals with ASD [13,87]. This journey will face numerous obstacles, including scientific challenges, public health and safety concerns, regulatory drug policies, economic implications, ethical considerations related to off-label and unregulated medication use, and societal perceptions surrounding changes in drug regulations and the approval of new, unconventional treatments. Progressing forward will undoubtedly prove to be extremely challenging.

### 2.6. Mescaline

The first recorded use of mescaline as a psychedelic agent was by Ernst Späth, a German pharmacologist. Späth, it is reported, used peyote regularly from 1919 to 1922 [124]. While skeptical at first, Späth was won over by the effects he experienced and gave his thoughts on its potential. He found that the substance considerably altered his mode of thinking, his sense of time and space, visual perception, and even tactile sensations. Späth also noticed that the experience could be affected by a person’s physiological condition and environmental context [125]. Peyote, scientifically known as *Lophophora williamsii*, is a type of cactus containing several biologically active alkaloids, including pellotine, anhalonidine, hordenine, and mescaline [126]. It is said that the long-term side effects of the drug are like those of LSD, i.e., abnormal flashbacks may affect the user many years after the cessation of usage. There are also psychological effects of using mescaline, e.g., the drug may prompt psychotic episodes or suicidal depression in individuals who have a pre-existing vulnerability to such conditions [127,128]. Furthermore, considerable damage to the transmission of normal brain messages—particularly those of serotonin—may wreck a person’s thought processes and affect any or all of a person’s senses [129,130]. Mescaline is typically taken from early to middle adulthood, and there is no evidence to suggest that it is physically addictive. Culturally, the use of mescaline often leads to a deeply spiritual experience that may be transformative [131,132,133]. Medical applications of mescaline, while not extensively significant, are accompanied by unverified assertions suggesting potential value in addressing mental or emotional issues [15,18,128,134]. Mescaline was specifically singled out by the United Kingdom’s Misuse of Drugs Act 1971 and made illegal to possess or distribute without a license [135]. Penalties include a substantial fine and/or imprisonment. Mescaline has been scheduled within Class A of the Misuse of Drugs Regulations since the 1990s [136]. This places it in the same category as crack cocaine and heroin. However, the law allows licensed possession, supply, and use for legitimate purposes, although these are few and difficult to obtain.

### 2.7. Salvia divinorum

*Salvia divinorum*, a psychoactive herb, contains the key non-alkaloid psychoactive compound salvinorin A, making it unique among other psychoactive plants. Salvinorin A, the psychoactive element found in Salvia, is the most potent naturally occurring hallucinogen, surpassing psilocybin [131,137,138,139,140,141,142]. Unlike most natural hallucinogens like DMT, psilocybin, and mescaline, Salvinorin A is a terpenoid and stands out as a non-alkaloid hallucinogen (Table 1). This structural difference translates to a distinct experience compared to other hallucinogens, often described as dissociative [143]. Our understanding of Salvia, particularly its effects and risks for people with ASD, remains limited due to minimal research.

### 2.8. 2C-B (4-Bromo-2,5-dimethoxyphenethylamine)

2C-B is structurally related to mescaline [132,133] and has been studied for its potential effects on social behavior and anxiety [144,145,146,147,148,149]. There is limited clinical evidence on the effects of 2C-B; however, a case report has been published on the effects of oral 2C-B ingestion in a healthy individual. On the Beck Depression Inventory (BDI) and the Generalized Anxiety Disorder 7-item scale (GAD-7), which are used to assess the level of depression and anxiety, the individual’s scores were in the “minimal depression” and “minimal anxiety” categories a day after ingestion of 2C-B [147]. The case report noted that the individual experienced a subjective feeling of being “healthy,” “balanced,” and “without fear” following ingestion. However, it is unclear how well these results can be extrapolated to the potential effects of 2C-B on individuals with ASD. It is known that 2C-B acts as a partial agonist at the 5-HT_2A_ and 5-HT_2C_ serotonin receptors [150,151]. This may lead to increased heart rate and blood pressure, and risky elevations in serotonin levels [145]. Chronic use of 2C-B is also known to lead to tolerance, meaning that larger doses of the drug are required to achieve the same effect. Tolerance to 2C-B develops rapidly, and it takes about a week to return to baseline sensitivity. The long-term effects of 2C-B are not yet fully understood. It is unclear whether the drug is addictive. The use of 2C-B for individuals with ASD remains unknown.

### 2.9. Myristicin

Myristicin is an organic compound that is found in nutmeg (*Myristica fragrans*). It is most used as a flavoring agent. It is also used in the preparation of various types of soap. Myristicin is used in traditional treatments for paralysis, joint pain, and various other ailments. It has been shown to have antimicrobial, antiproliferative, and anti-inflammatory activities [152]. Most importantly, it is used as a psychoactive substance. It is reported that Myristicin has certain psychoactive properties. Perhaps because of these properties, the use of this compound as a flavoring agent in the food industry is limited in many countries. This compound is reported to cause feelings of happiness and wellness. Early research has shown that rat livers can metabolize myristicin into MMDA [153], a known amphetamine. Interestingly, Myristicin was also shown to have a hepatoprotective effect [154]. Given its psychoactive effect, its potential use in the management of ASD symptoms is promising. No clinical evidence or studies have been done to assess this aspect of myristicin.

## 3. Understanding Autism Spectrum Disorder

Autism Spectrum Disorder represents a multifaceted developmental condition profoundly influencing communication and behavioral patterns. Its designation as a “developmental disorder” underscores its typical manifestation in the first 2 years of life. Defined by the Diagnostic and Statistical Manual of Mental Disorders (DSM-5) [155], ASD encompasses enduring challenges in social communication and interaction, along with restricted and repetitive behavioral tendencies. Such diagnostic parameters necessitate observable deficits in these core domains for an ASD diagnosis. The National Institute of Mental Health (NIMH) actively investigates the prevalence and multifactorial influences contributing to ASD, aiming to elucidate its complex etiology. The Centers for Disease Control and Prevention (CDC) established the Autism and Developmental Disabilities Monitoring (ADDM) Network to track autism spectrum disorder and cerebral palsy (CP) in children across the United States. This unique collaboration offers a wealth of information about these developmental conditions. Recent findings show that in 2020, around 1 in 36 eight-year-olds within the network had ASD. Importantly, racial and ethnic disparities in identification appear to be narrowing, with Black, Hispanic, and Asian or Pacific Islander children showing higher diagnosis rates compared to White children. However, boys were still nearly four times more likely to be diagnosed with ASD than girls [156]. Furthermore, while the DSM-5 furnishes a comprehensive framework for ASD diagnosis, ongoing updates that reflect evolving research findings and clinical insights remain imperative. Each revision of the DSM integrates contemporary knowledge to refine diagnostic criteria, ensuring alignment with prevailing scientific understanding and fostering universal diagnostic practices among clinicians and researchers alike.

### 3.1. Current Management Approaches for Autism Spectrum Disorder

Current management approaches for ASD encompass a diverse array of behavioral, educational, medical, and therapeutic interventions designed to enhance the quality of life for those affected by this condition. In the United States, where approximately 1 in 44 children are diagnosed with ASD, the importance of effective management strategies is critical for both families and healthcare providers [157,158,159]. While no known cure exists for ASD, early intervention and individualized management plans significantly improve communication, social abilities, and daily functioning. Applied Behavior Analysis (ABA) is one of the most established interventions [158]. This data-driven approach focuses on modifying behaviors through reinforcement and has extensive empirical support, highlighting its effectiveness in promoting adaptive behaviors while reducing maladaptive ones. This approach makes ABA a foundational therapy in ASD management [160]. Emerging strategies, such as Positive Behavior Support (PBS) and technology-assisted interventions, are also gaining prominence, emphasizing improvements in communication skills and social engagement through innovative methods [161,162]. Medical interventions for ASD typically include pharmacological options, mainly for managing co-occurring symptoms like anxiety, mood disorders, and irritability. Notably, the FDA has approved risperidone and aripiprazole for specific behavioral symptoms associated with ASD [163,164]. However, non-pharmacological therapies, including speech and language therapy, cognitive-behavioral therapy, and dietary adjustments, are also integral to creating a comprehensive treatment plan tailored to the individual’s needs [165,166,167,168,169]. Controversies surrounding ASD management often focus on balancing the acceptance of neurodiversity with interventions aimed at improving functioning and quality of life. The neurodiversity movement emphasizes the value of cognitive diversity, challenging approaches that seek to “normalize” autistic behaviors solely. This debate highlights the importance of a personalized, collaborative approach to managing ASD, one that recognizes individual differences while aiming for practical improvements in daily living.

### 3.2. Types of Management Approaches

Current management strategies for ASD primarily involve established behavioral and educational interventions, such as Applied Behavior Analysis. ABA aims to understand and modify observable behaviors by analyzing their antecedents and consequences of behaviors. Its strong empirical backing demonstrates its ability to increase adaptive behaviors and reduce maladaptive ones in individuals with ASD [157,158,159]. ABA’s tailored approach, based on continuous data collection on target behaviors [170] allows interventions to evolve as the individual progresses. Integrating ABA into daily routines across various settings, including home and school, is also crucial for skill acquisition [171].

### 3.3. Medical and Therapeutic Interventions

#### 3.3.1. Medical Treatment

Pharmacological treatments are often utilized to manage symptoms associated with autism spectrum disorder, such as anxiety and mood disorders. Common medications include antipsychotics, antidepressants, stimulants, and anxiolytics. For example, the FDA has approved risperidone and aripiprazole specifically for treating irritability and aggression in children with ASD [172,173,174,175,176]. Selective serotonin reuptake inhibitors (SSRIs) can help manage anxiety and depression, while stimulants are effective in improving focus and reducing hyperactivity. Close physician oversight is essential, as these medications can have side effects like weight gain and metabolic issues. Experimental therapies, such as intranasal oxytocin and glutamatergic agents, are also under investigation for their potential to address ASD symptoms [177]. Additionally, complementary treatments, including melatonin, omega-3 fatty acids, and probiotics, are used by some individuals with ASD, though their efficacy remains variable [178].

#### 3.3.2. Pharmaceutical Interventions

Pharmaceutical interventions for ASD often aim to manage core symptoms, such as repetitive behaviors, social and communication difficulties, and challenges in adapting to different environments [170]. These interventions typically involve two main drug classes: antipsychotics and antidepressants. Antipsychotics work by blocking dopamine effects in the brain, which can reduce repetitive behaviors, irritability, and agitation. Antidepressants, which increase levels of neurotransmitters like serotonin, help improve mood, reduce anxiety, and enhance flexibility in adjusting to different situations [179,180,181,182]. However, the effectiveness of these medications varies widely among individuals, and they carry potential side effects. For example, antipsychotics may cause sleepiness, weight gain, movement disorders, and hormone disturbances, while some antidepressants may increase the risk of self-harm and suicidal thoughts [179,180,183,184,185,186]. Therefore, decisions regarding pharmaceutical use must involve careful assessment of each individual’s needs, balancing potential benefits with risks. Ongoing monitoring and collaboration among healthcare providers, caregivers, and patients are necessary to ensure safe and optimal medication use. Regular adjustments to personalized treatment plans enable a response to evolving patient needs and the latest research insights. Large-scale, controlled studies are still needed to better understand these medications’ effects on developing brains in young individuals with ASD and to identify those who may benefit most based on genetic and neurobiological profiles. Such research could advance precision medicine in ASD, aiming to tailor treatment approaches to each individual’s unique characteristics and needs.

## 4. Psychedelics and Their Potential for Autism Spectrum Disorder Management

**Figure 1:**
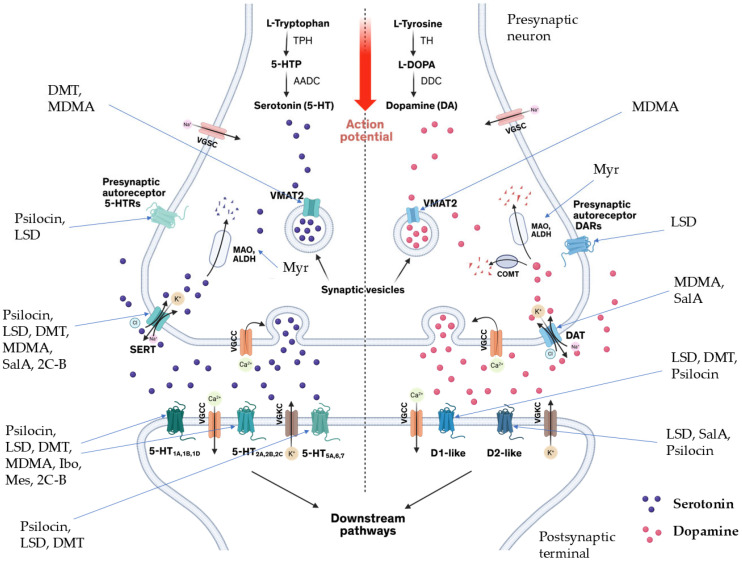
Psychedelics and their influence on synaptic plasticity of serotonergic and dopaminergic neurons, with implications for autism therapy. Most psychedelics act primarily as agonists at the serotonin 5-hydroxytryptamine 2A receptor (5-HT_2A_ receptor), a pivotal element of the serotonergic system that modulates mood, perception, and cognition. Additionally, activation of other serotonin and dopamine receptors contributes to the psychoactive and behavioral effects of these substances. In presynaptic neurons, psychedelics influence serotonin and dopamine transporters, autoreceptors, vesicular monoamine transporters, and neurotransmitter release. Blue arrows indicate potential targets of psychedelic action in the CNS. Abbreviations: lysergic acid diethylamide (LSD), dimethyltryptamine (DMT), 3,4-methylenedioxymethamphetamine (MDMA), ibogaine (Ibo), mescaline (Mes), salvinorin A (SalA), 4-bromo-2,5-dimethoxyphenethylamine (2C-B), myristicin (Myr), 5-hydroxytryptamine (5-HT), tryptophan hydroxylase (TPH), 5-Hydroxytryptophan (5-HTP), aromatic L-amino acid decarboxylase (AADC), serotonin transporter (SERT), tyrosine hydroxylase (TH), L-3,4-dihydroxyphenylalanine (L-DOPA), DOPA decarboxylase (DDC), dopamine transporter (DAT), monoamine oxidase (MAO), aldehyde dehydrogenase (ALDH), catechol-O-methyltransferase (COMT), vesicular monoamine transporter 2 (VMAT2), voltage-gated sodium channel (VGSC), voltage-gated calcium channel (VGCC), voltage-gated potassium channel (VGKC), Central Nervous System (CNS). Serotonin receptor subtypes implicated in psychedelic effects: 5-HT_1A_, 5-HT_1B_, 5-HT_1D_, 5-HT_2A_, 5-HT_2B_, 5-HT_2C_, 5-HT_5A_, 5-HT_6_, 5-HT_7_, presynaptic 5-HT autoreceptors. Dopamine receptor subtypes implicated in psychedelic effects: D1-like (D_1_ and D_5_), D2-like (D_2_, D_3_, D_4_), presynaptic DA autoreceptors. (Created in BioRender. Demirkhanyan, L. (2026) https://BioRender.com/5oyrc5l).

Since the discovery of lysergic acid diethylamide by Dr. Albert Hofmann in 1943 [187,188], numerous psychedelic substances have been investigated for their pharmacotherapeutic effects [189,190,191,192]. Defined by their ability to induce profound alterations in sensory perception, mood, and cognitive processes, these drugs interact with a diverse range of pharmacological targets (Figure 1). While their primary action is often attributed to the serotonin 5-HT_2A_ receptor, their clinical utility in ASD management stems from a broader modulation of multiple serotonergic and dopaminergic pathways that regulate memory and sensory perception (Table 3).

### 4.1. Psilocybin in Autism Management

The therapeutic potential of psilocybin has received significant scientific attention in recent years, particularly following promising clinical trials for conditions such as treatment-resistant depression and end-of-life anxiety. Recent research has highlighted psilocybin’s potential beyond traditional therapeutic domains, including studies exploring its effects on mental health and social engagement among autistic adults [193]. A recent survey of autistic participants demonstrated that a single impactful psychedelic experience often leads to significant improvements in mental health and social interaction [12]. These findings expand our understanding of psilocybin’s potential applications, highlighting the importance of controlled environments to maximize benefits while minimizing risks. Together, these insights reinforce the broad therapeutic potential of psilocybin while underscoring essential considerations for its safe implementation. Psilocybin functions as a prodrug, converting to psilocin, the active compound responsible for its psychoactive effects. Psilocin acts primarily as a partial agonist at the 5-HT_2A_ receptor, producing its characteristic psychedelic effects. Following ingestion, psilocybin undergoes rapid dephosphorylation in the stomach to form psilocin, which then crosses the blood–brain barrier. Psilocin’s effects peak around 120 min post-ingestion, with a half-life of approximately 108 min. The compound is metabolized by hepatic enzymes and excreted via renal and biliary pathways [194]. Clinical trials initially implemented weight-based dosing for psilocybin, ranging from 0.2 to 0.4 mg/kg. More recent protocols have shifted towards a fixed-dose approach, commonly around 25 mg, which has demonstrated consistent therapeutic effects. Psilocybin administration should be accompanied by structured psychotherapy sessions, encompassing pre-treatment preparation, supportive in-session guidance, and post-treatment integration. A controlled setting and patient preparedness—often described as “set and setting”—are crucial for optimizing therapeutic outcomes and minimizing adverse experiences [195].

Currently, the PSILAUT trial (NCT05651126), sponsored by King’s College London, investigates psilocybin’s effects on brain function in adults with and without ASD. Using a double-blind, placebo-controlled design, the study examines neural and subjective responses to a single dose of psilocybin (COMP360) with advanced imaging and electrophysiological methods, including fMRI, EEG, and magnetic resonance spectroscopy (MRS) [196]. Primary measures assess changes in resting-state brain activity, sensory-evoked potentials, and brain connectivity, while secondary measures focus on neurotransmitter balance in the brain’s excitatory and inhibitory systems. By exploring how psilocybin affects neural processing in neurotypical versus ASD individuals, PSILAUT aims to inform potential therapeutic applications for ASD and contribute to broader research on psychedelics in mental health treatment. Another recent study explored the effects of a single impactful psychedelic experience on mental health and social engagement among autistic adults. The online survey, conducted with 233 autistic participants, assessed changes in psychological distress, social engagement, and psychological flexibility. Results revealed that a majority of participants experienced reductions in psychological distress (82%) and social anxiety (78%), alongside increased social engagement (70%). However, approximately 20% reported negative outcomes (Table 4), such as increased anxiety, highlighting the need for careful patient selection and controlled settings for psychedelic therapy in this population. Psychological flexibility emerged as a substantial predictor of reduced distress, suggesting a potential therapeutic mechanism by which psilocybin may enhance resilience and adaptability in autistic individuals [197]. As the therapeutic use of psilocybin grows, it is essential for health care professionals (HCPs) to acquire foundational knowledge of its pharmacology, therapeutic applications, and safety requirements. With the potential to alleviate severe psychiatric symptoms and foster social engagement, psilocybin shows promise across a diverse patient population, including individuals with autism. However, the necessity for controlled settings, proper dosing, and a comprehensive safety assessment cannot be overstated. An informed HCP approach will not only improve patient outcomes but also support the development of clinical and regulatory frameworks, ensuring that psilocybin’s therapeutic benefits are accessible, safe, and ethically managed.

### 4.2. LSD in Autism Management

Research into the potential use of lysergic acid diethylamide for managing ASD is still in its nascent stages. However, initial investigations suggest that LSD may offer therapeutic benefits by enhancing social interactions, reducing anxiety, and improving sensory processing among individuals with autism [12]. The neurochemical effects of LSD, particularly its impact on neural plasticity and social cognition, present intriguing possibilities for addressing some of the core challenges associated with ASD [198]. LSD was first synthesized in 1938 and initially explored for various medical uses before its psychoactive properties were recognized. Despite extensive research on LSD during the 1950s and 1970s, much of this work did not adhere to contemporary scientific standards. Consequently, research efforts halted when LSD was prohibited in many countries [187,199,200,201]. Recent years have witnessed a resurgence of interest, with researchers aiming to reassess its potential therapeutic applications in various psychiatric conditions, including autism. Early findings indicate that LSD may enhance social capabilities and alleviate anxiety in individuals with autism, although scientific evidence remains limited and requires further validation. The complexities of designing double-blind, placebo-controlled studies pose significant ethical challenges, especially when considering the unique vulnerabilities of individuals with autism [202]. The historical context of LSD research also presents limitations, as many earlier studies are now considered outdated and lacking in methodological rigor [202]. The exploration of LSD as a treatment for autism involves considerable ethical considerations, particularly regarding informed consent and the ability of individuals with autism to engage in the research process meaningfully. Given the profound effects of LSD on perception and cognition, careful monitoring of psychological outcomes is essential. Preliminary evidence suggests that LSD may have a positive impact on social interaction, communication skills, and anxiety levels in individuals with autism. Specifically, studies indicate that psychedelics like LSD might stimulate serotonin receptors that are implicated in autism, potentially facilitating enhanced social engagement and reduced anxiety during social interactions [203]. Despite the promising findings, caution is warranted when considering the use of LSD for autism management. The current evidence largely stems from anecdotal reports and limited clinical studies, necessitating further rigorous research to establish the safety and efficacy of LSD as a treatment modality. Moreover, experts caution against viewing psychedelics as a cure for autism, emphasizing that psychiatric interventions should focus on enhancing quality of life rather than altering the underlying neurodevelopmental characteristics of autism. The use of LSD in the treatment of ASD poses several safety and side effect considerations that require thorough evaluation. Early clinical trials in the 1960s and 1970s revealed both beneficial outcomes and significant adverse effects in children with ASD who were administered psychedelic treatments. Positive behavioral changes included improved mood, sociability, and enhanced communication abilities (Table 4), while negative effects encompassed mood swings, anxiety, seizures, and increased aggression [204]. This duality underscores the importance of conducting further studies to assess the overall safety and efficacy of LSD for individuals with ASD. Individuals with autism may experience heightened sensitivity to sensory stimuli, which can lead to overwhelming feelings of anxiety or panic when exposed to the intense sensory experiences induced by LSD. Furthermore, the altered states of consciousness produced by LSD may complicate the processing and integration of complex information, potentially exacerbating the challenges faced by those with ASD. In addition to psychological risks, there are pharmacological concerns related to the use of LSD. Its hallucinogenic properties are mediated by interactions with various serotonin receptors, leading to effects such as altered perception and emotional responses (Figure 1). As with any psychoactive substance, careful monitoring by experienced healthcare professionals is critical to ensure patient safety, especially considering the possible side effects associated with LSD, such as increased heart rate, anxiety, and the potential for a “bad trip,” which could have lasting psychological impacts.

### 4.3. DMT in Autism Management

Recent studies suggest that disruptions in the pineal gland and its melatonin production may play a role in ASD, particularly given that melatonin regulates the body’s circadian rhythms and sleep–wake cycle. Notably, sleep disturbances and low melatonin levels are commonly observed in ASD, pointing to potential pineal dysfunction as a contributing factor to autism’s neurobiological profile [205]. In addition to melatonin, recent research has highlighted the potential involvement of endogenous dimethyltryptamine (DMT), another compound synthesized in the pineal gland. Autism is associated with atypical neuroplasticity, including cortical overgrowth and irregular dendritic spine development. It has been proposed that elevated DMT levels, resulting from dysregulation in the pineal gland, could contribute to these neuroplastic changes [206]. This DMT hyperactivity hypothesis aligns with observations of neurodevelopmental abnormalities in ASD, suggesting that DMT may influence neuroplasticity in ways that impact social cognition and adaptive behavior. Given these findings, researchers propose that therapeutic strategies to balance DMT and melatonin production might offer new avenues for autism treatment. Exogenous melatonin administration and monitored light exposure are two potential interventions aimed at restoring pineal function. Additional research is needed to test the DMT hypothesis and clarify whether DMT dysregulation indeed contributes to the pathophysiology of ASD. Investigating this further could establish a biological foundation for autism therapies targeting the pineal gland [205]. This perspective underscores the importance of studying DMT’s role in neurodevelopment and ASD, paving the way for biologically informed interventions that may improve quality of life and adaptive functioning in autistic individuals.

### 4.4. MDMA in Autism Management

MDMA has emerged as a promising pharmacological agent with potential applications in the management of ASD, owing to its unique effects on social cognition and empathy. Despite being termed an “empathogen” for its prosocial effects, identifying specific mechanisms underlying MDMA’s impact has proven challenging. A recent study paired the social transfer of pain and analgesia with neuropharmacological and optogenetic techniques in mice, demonstrating that MDMA enhances empathy-like behaviors via serotonin (5-HT) signaling in the nucleus accumbens (NAc). Further, MDMA or optogenetic stimulation of NAc 5-HT pathways restored empathy deficits in an ASD mouse model, specifically Shank3-deficient mice, indicating serotonin’s role in MDMA’s empathogenic effects [207]. In clinical settings, MDMA-assisted psychotherapy has shown promise for alleviating social anxiety in autistic adults. A blinded, placebo-controlled pilot trial investigated MDMA’s effects on social fear and avoidance in autistic adults with severe social anxiety. Results indicated significant improvements in social anxiety scores (*p* = 0.037), with sustained benefits observed at a six-month follow-up. This supports MDMA’s potential as a safe and effective therapy for social anxiety in ASD, with rapid and durable outcomes [82]. MDMA’s impact on social behavior is being explored across multiple conditions, but most of the clinical research has historically focused on its use in posttraumatic stress disorder [208]. Recently, however, attention has shifted to additional psychiatric applications, including autism-related social anxiety and alcohol use disorder. A systematic review [193] also highlighted MDMA’s effectiveness in enhancing social behavior in ASD, with controlled trials reporting improved sociability and interpersonal closeness (Table 4), outcomes linked to MDMA’s activation of serotonin and oxytocin pathways. The social challenges associated with ASD, including social-anxiety-related avoidance and difficulties in social cognition, make MDMA a compelling adjunct to current ASD therapies. In addition to altering social behaviors, MDMA may directly target neurological processes involved in social reward sensitivity, anxiety reduction, and social approach behaviors [209]. Although further research is warranted, these findings suggest that MDMA may play a unique role in managing ASD, offering new avenues for improving social functioning in affected individuals. MDMA’s capacity to enhance empathy, alleviate social anxiety, and foster positive social interactions holds significant promise for the management of autism. While current studies are still preliminary, these findings suggest that MDMA use could become a transformative approach for addressing social challenges in autism. Further research, including larger controlled trials, is warranted to better understand and confirm these effects.

### 4.5. Ibogaine in Autism Management

In recent years, ibogaine has gained attention in the context of autism management, with proponents suggesting it may offer novel therapeutic benefits for individuals with ASD. The compound’s complex interactions with neurotransmitter systems and its potential to enhance neuroplasticity have fueled interest in its application for various mental health conditions, including autism [103]. Despite this, the use of ibogaine in autism therapy is fraught with controversy and ethical concerns, particularly in relation to established interventions like Applied Behavior Analysis. Research indicates that ibogaine may impact cognitive and emotional functioning, which are often challenged by individuals on the autism spectrum [210]. However, the therapeutic application of ibogaine is hampered by significant regulatory hurdles, including its classification as a Schedule I substance in the United States, which restricts legal access and comprehensive research into its efficacy and safety [211,212,213,214,215,216]. The existing literature on ibogaine’s use for autism management highlights both its potential benefits and considerable risks, including adverse effects such as cardiac complications and psychological disturbances [217]. This dichotomy underscores the pressing need for more rigorous scientific inquiry to evaluate ibogaine’s safety profile and therapeutic effectiveness for ASD. Controversy surrounds the ethical implications of using ibogaine in autism management, particularly regarding the adequacy of existing treatments and the involvement of autistic individuals in therapy development. Critics argue that traditional approaches like ABA may not align with the best interests of autistic individuals, raising questions about informed consent and autonomy in treatment decisions [218]. Additionally, there are concerns that the increasing interest in alternative therapies, including ibogaine, may lead families to pursue unvalidated treatments out of desperation, which could potentially cause harm [219]. This complex landscape necessitates ongoing dialog and research to ensure that any therapeutic application of ibogaine respects ethical principles and prioritizes the well-being of individuals with ASD. Emerging research has also begun to explore ibogaine’s ability to promote neuroplasticity, which may contribute to its therapeutic effects in mental health disorders. Evidence suggests that ibogaine may enhance levels of brain-derived neurotrophic factor (BDNF; Table 3), a key protein linked to improved mood and cognitive function [220,221,222]. Additionally, preclinical studies have indicated anxiolytic properties, which could further support its application in treating anxiety-related symptoms. Despite the potential benefits of ibogaine, the current body of research highlights the necessity for controlled clinical trials and more extensive studies to firmly establish its safety and efficacy in clinical settings. This includes addressing concerns over its adverse effects and ensuring a comprehensive evaluation of its therapeutic mechanisms and outcomes across diverse populations.

### 4.6. Mescaline in Autism Management

Despite its long history of use, research on mescaline’s therapeutic potential has been limited (Figure 1). Preliminary anecdotal evidence suggests that mescaline may offer mental health benefits, particularly for conditions such as depression, anxiety, and PTSD [223]. A recent survey involving 452 respondents indicated that a significant portion (68–86%) of those with histories of mental health issues reported subjective improvements following memorable mescaline experiences [223]. Interest in mescaline has grown in the context of psychedelic research, with studies exploring its safety profile and potential therapeutic applications in modern medicine. However, a comprehensive understanding of its safety and efficacy remains under investigation.

### 4.7. Salvia divinorum in Autism Management

The activation of kappa-opioid receptors (KORs) by salvinorin A occurs in brain regions associated with perception, mood regulation, and the stress response, including the cortex, hippocampus, and amygdala. This interaction can produce dissociative effects, leading to alterations in sensory perception, self-awareness, and one’s experience of external reality. Research suggests that salvinorin A may influence the dopaminergic and glutamatergic systems, although the exact mechanisms of these effects remain under investigation [224,225,226,227,228]. There is a growing interest in salvinorin A’s potential for treating conditions like substance addiction and mental health disorders. KOR agonists like salvinorin A may impact reward pathways in the brain, potentially reducing the reinforcing effects of addictive substances [224]. Early studies indicate possible therapeutic applications in managing resistant conditions, including post-traumatic stress disorder, depression, and anxiety [229]. While *Salvia divinorum* shows promise, its health benefits remain underexplored, warranting more scientific scrutiny to confirm these therapeutic claims [226]. Current research is examining the potential role of *Salvia divinorum* in managing mood disorders and related symptoms in individuals with autism. The unique psychoactive properties of salvinorin A could influence neurological pathways associated with anxiety, depression, and chronic pain—issues often prevalent in autism spectrum disorder populations. Researchers continue to investigate these interactions to better understand the benefits and risks of using *Salvia divinorum* in autism management [224,225,230].

### 4.8. 2C-B in Autism Management

4-Bromo-2,5-dimethoxyphenethylamine, commonly referred to as 2C-B, is a synthetic psychedelic compound that belongs to the phenethylamine family and has garnered attention for its potential therapeutic applications, particularly in managing ASD. This compound, characterized by its agonistic activity at the 5-HT_2A_ serotonin receptor, demonstrates unique neuroplasticity-inducing properties, positioning it as a candidate for innovative treatment modalities in neuropsychiatric conditions, including ASD [145,146]. The exploration of 2C-B in the context of autism management is still in early stages, but preliminary studies suggest that it may help alleviate certain symptoms associated with the disorder, such as social interaction difficulties and sensory processing issues [231]. This potential therapeutic benefit has led to discussions about the implications of incorporating psychedelics into mainstream psychiatric care. However, this topic is not without controversy; concerns regarding the safety, efficacy, and long-term effects of 2C-B remain significant, particularly given its classification as a controlled substance in many jurisdictions worldwide [218,232]. Notably, while 2C-B offers promising avenues for treatment, its efficacy relative to established pharmacological and behavioral interventions for ASD warrants further investigation. Conventional treatments typically involve selective serotonin reuptake inhibitors and behavioral therapies, which have demonstrated substantial empirical support. As such, the integration of 2C-B into therapeutic practices must be approached cautiously, with comprehensive clinical trials needed to establish safety guidelines and efficacy protocols [233,234,235]. As research progresses, the ethical considerations surrounding the use of psychedelics like 2C-B in clinical settings will also become increasingly important. Stakeholder engagement, including perspectives from individuals with autism and their families, is crucial for shaping responsible treatment frameworks that prioritize patient safety and therapeutic integrity [231]. The ongoing inquiry into the role of 2C-B in autism management could pave the way for transformative advancements in the understanding and treatment of ASD and related neuropsychiatric disorders.

### 4.9. Myristicin in Autism Management

While myristicin is primarily recognized for its hallucinogenic effects when consumed in large quantities, it has also sparked interest within the context of autism spectrum disorder management, raising questions about its efficacy and safety as a treatment option [236,237]. The psychoactive effects of myristicin, which can include hallucinations, euphoria, and anxiety, often resemble those of cannabinoids like THC, but can also lead to negative experiences such as paranoia and confusion [237,238]. Due to its unpredictable effects and potential for severe outcomes at high doses, including addiction, the use of myristicin as a therapeutic agent in ASD management remains controversial. The rising interest in alternative treatments, including myristicin, reflects a broader trend among families seeking non-traditional approaches to managing autism-related challenges, amidst concerns about the limitations and side effects of conventional therapies [239]. In the realm of ASD, treatment strategies typically include behavioral interventions and pharmacological options. However, the effectiveness of these approaches, especially alternative therapies like myristicin, is often debated due to inconsistent findings in research. Many studies investigating pharmacological treatments for ASD have yielded mixed results, necessitating further investigation to clarify the potential benefits and risks of incorporating substances like myristicin into treatment regimens. The discussion surrounding myristicin’s role in autism management highlights significant methodological challenges within research on ASD treatments. Issues such as small sample sizes and the lack of diverse participant groups often complicate the ability to ascertain the safety and effectiveness of various interventions, including myristicin [240,241]. As the interest in alternative therapies continues to grow, it is crucial for families and healthcare providers to navigate these options with caution, emphasizing the importance of empirical evidence and professional guidance in treatment decisions.

## 5. Emerging Clinical Evidence on Psychedelics and Autism Spectrum Disorder

Of the few small-scale, preliminary clinical studies conducted to date, results have been mixed. However, there is emerging evidence suggesting that psychedelics may have a positive impact on core symptoms and associated features of Autism Spectrum Disorder—at least for some individuals. A case series of twelve patients with treatment-resistant ASD who received a single dose of the psychedelic MDMA as part of a pilot study was published in 2018. A significant reduction in autistic social impairment and caregiver-rated repetitive behaviors and sensory abnormalities was reported in the 11 individuals who completed the course of MDMA-assisted therapy, as measured between 3 and 6 months post-treatment. The improvements in social function were sustained at 9 months post-treatment, along with evidence of improved quality of life in these patients. However, the limitations of the case series (such as lack of control group, relatively low number of participants, potential bias in the outcome measures, and the need for replication with larger, placebo-controlled studies), as well as the ambiguity in the exact mechanisms and duration of effects, warrant caution in interpreting the results. Let’s consider the fact that the drug MDMA is already known to have abuse potential and harmful effects overall, as categorized by the US Controlled Substances Act. There is another ongoing study on the use of psilocybin in treating social withdrawal and avoidance in children with ASD, though it has not yet published its findings. It is an open-label pilot study in which all participants receive the treatment under investigation, typically without a control group, thereby limiting the reliability of the results. It is interesting to note that individuals’ levels of sensory overload to certain stimuli were found to drop when they were shown images while under the influence of psilocybin, in addition to a decrease in recognition of negative facial expressions. This might suggest a potential capability of psilocybin to ameliorate altered sensory perception and social deficits in ASD, but more clinical evidence would be necessary. In any case, it is vital for researchers to keep in mind that an ‘optimal’ intervention for ASD is expected to help improve not only the core symptoms, such as social communication and interaction difficulties and repetitive behaviors, but also the associated phenotypes that could affect one’s everyday life as well. Any abnormal doctor’s shopping, drug ingestion, or serious involvement should be reported and investigated. Well-powered, randomized, placebo-controlled clinical trials need to be designed and conducted to provide stronger evidence, preferably involving more than one type of psychedelic substance. These studies will highly facilitate the process of initiating the central authoritative approval of certain psychedelics as clinical treatments for ASD.

### 5.1. Mechanisms Underpinning Psychedelic Effects on ASD Symptoms: An Expert Overview

#### 5.1.1. The Intense World Theory and Cellular Microcircuitry in ASD

The “intense world theory” provides a prominent hypothesis for understanding the symptomatic manifestations of ASD. This theory posits that ASD is characterized by hyperfunctioning of neural microcircuitry—networks of interconnected neurons at the cellular level that process and integrate signals from the nervous system. Hyperactive microcircuitry may contribute to heightened sensitivity to environmental stimuli and cognitive overload, hallmark features of ASD. Emerging research suggests that psychedelics could mitigate these disruptions by modulating the serotonergic system, particularly through activation of the 5-HT_2A_ receptor. This receptor activation results in downstream effects, including desensitization of 5-HT_2C_ receptors, leading to increased glutamate release. Glutamate, a key excitatory neurotransmitter, promotes greater neuroplasticity by reducing restrictive serotonin-driven signaling, which may potentially alleviate the neural rigidity observed in ASD [52,61,79,150,151,242].

#### 5.1.2. Serotonergic Modulation by Psychedelics

The serotonergic system, encompassing serotonin pathways, receptors, and neurotransmitter dynamics, plays a pivotal role in the mechanism of psychedelics relevant to ASD. Psychedelics primarily act on 5-HT_2A_ receptors [61,63,70,243], which are implicated in processes of synaptic plasticity and network reorganization. Activation of these receptors promotes glutamate release, triggering cascades associated with neuroplasticity. Unlike conventional treatments such as SSRIs, which enhance serotonin availability but lack robust effects on plasticity, psychedelics uniquely stimulate dendritic spine growth and synaptic remodeling in the prefrontal cortex [244]. Since this brain region is critical for social cognition and decision-making, its dysfunction in ASD underscores the therapeutic potential of psychedelics (Table 3). However, chronic exposure to psychedelics requires careful scrutiny, as some studies suggest receptor downregulation over time, necessitating further research into optimal dosing regimens and long-term safety [245,246,247].

#### 5.1.3. Neuroplasticity and Psychedelic-Assisted Therapy

Neuroplasticity, the brain’s capacity to reorganize itself through synaptogenesis and neuronal growth, is integral to learning, memory, and adaptation to stress. Dysfunctional plasticity in ASD may underlie difficulties in adapting to environmental and social cues [248]. Psychedelic-assisted therapies, leveraging agents such as ketamine (a non-classical psychedelic with shared mechanisms), have demonstrated rapid and durable effects in enhancing plasticity and ameliorating symptoms of neuropsychiatric disorders [249]. Clinical studies in major depressive disorder highlight the potential of psychedelics to induce rapid symptom relief, outperforming traditional treatments like SSRIs in both onset speed and efficacy [250]. These insights suggest a promising avenue for treating ASD, where enhanced plasticity could mitigate cognitive and behavioral symptoms. Importantly, integrating psychedelics with psychotherapy ensures that neuroplastic changes are directed toward therapeutic outcomes [251].

## 6. Safety, Ethical Considerations, and Study Design

The therapeutic use of psychedelics for ASD carries significant safety and ethical implications that necessitate a rigorous framework to protect participants and ensure scientific validity [252,253]. While low doses may theoretically provide benefits such as improved mood and reduced stress, several critical concerns regarding the use of Schedule I substances in vulnerable populations must be addressed [254].

### 6.1. Safety Considerations and Risks

Because psychedelics are primarily studied in animal models, substantial gaps remain regarding their long-term safety in humans, particularly those with neurodevelopmental conditions [196]. Key safety concerns include:

• **Adverse Psychological Effects:** Psychedelics can elicit intense sensory distortions, paranoia, or “bad trips,” which may exacerbate the sensory sensitivities common in ASD [252]. Although rare, severe complications such as seizures or hallucinogen persisting perception disorder (HPPD) have been reported [252,253,255].
• **Impaired Autonomy and Vulnerability:** Individuals with ASD may face challenges in providing informed consent due to cognitive or communicative diversity [204,256]. There is also a heightened risk of undue influence or coercion, necessitating rigorous screening to ensure participant welfare.

### 6.2. Ethical Framework and Informed Consent

Ethical research in this field must prioritize the rights of the individual through robust, tailored consent processes. This involves simplifying complex medical information and incorporating caregiver support while respecting the participant’s autonomy [196]. Critics argue that it may be unethical to expose participants to potential harm if the research demands exceed a favorable risk-benefit balance. Additionally, the use of deception (e.g., active placebos) to preserve study validity is ethically justifiable only when alternative methodologies are infeasible, and approval is granted by an institutional review board.

### 6.3. Clinical Trial Design and Regulatory Oversight

The unique challenges of psychedelic research require careful study design to ensure reliability. Researchers must implement rigorous risk assessments, provide access to trained therapists, and maintain strict monitoring protocols to prevent therapeutic misconceptions—ensuring that caregivers and participants understand the experimental nature of the treatment [196,257].

Furthermore, a collaborative effort involving researchers, clinicians, and legislators is required to navigate the complex regulatory landscape of Schedule I substances. Comprehensive frameworks must ensure that practices align with professional and legal standards, particularly as research expands into sensitive areas like ASD management [256,257,258]. Balancing innovation with ethical rigor is vital to designing responsible and impactful clinical investigations.

## 7. Future Directions

### 7.1. Regulatory and Clinical Hurdles

The landscape of psychedelic research is significantly impacted by regulatory classifications. For instance, while the breakthrough therapy designation is intended to expedite drug development for serious conditions, the requirement for specific disease severity scales poses a challenge for ASD, given its highly individualized and varied manifestations [149]. Furthermore, the Drug Enforcement Administration (DEA) classification of substances like MDMA and psilocybin as Schedule I makes legal patient access and clinical administration difficult, even in research settings. Overcoming the barriers of registration and inspection processes is essential for clinicians to become experts in this emerging field. Nonetheless, continued clinical research and education provide a potential path forward to improve these systemic challenges.

### 7.2. Challenges in Psychedelic Research for Autism Spectrum Disorder

Emerging evidence suggests that psychedelics could enhance neuroplasticity and improve social functioning, potentially addressing some of the core challenges associated with ASD [204]. Early-stage studies indicate that psychedelics may facilitate the rewiring of neural circuits involved in social cognition and sensory integration, offering a novel therapeutic approach for ASD [196]. However, the field faces notable challenges, including ethical considerations, regulatory constraints, and the need for comprehensive clinical trials to establish the safety, efficacy, and long-term effects of these treatments [160]. The exploration of psychedelics for ASD also requires innovative research methodologies, such as the use of multimodal imaging and personalized therapeutic protocols, to better understand the nuanced effects of these compounds. Recent studies, such as the PSILAUT trial, aim to elucidate how psychedelics influence brain activity and social behavior in both autistic and non-autistic individuals [196]. These studies incorporate advanced imaging techniques like functional MRI and electroencephalography to assess neural changes and serotonin receptor dynamics under the influence of psychedelics [196]. Despite the promise of psychedelic therapies, several challenges must be addressed. Ethical considerations, such as obtaining informed consent from participants with varying cognitive abilities, are paramount. Many individuals with ASD may face difficulties understanding the risks and benefits of psychedelic interventions, underscoring the need for tailored consent processes and robust participant support systems. Additionally, regulatory barriers, including the scheduling of psychedelics as controlled substances, limit the accessibility and scalability of research [259]. Diversity and inclusivity in clinical trials remain critical issues, as historical biases in clinical research have often excluded marginalized populations. Ensuring diverse representation in psychedelic research is essential to improve the generalizability of findings and address the needs of all individuals with ASD [204]. Looking ahead, the successful integration of psychedelics into ASD treatment frameworks will depend on rigorous clinical trials, ethical oversight, and interdisciplinary collaboration. The development of standardized therapeutic protocols, combined with ongoing advancements in imaging and computational modeling, could provide deeper insights into the neurobiological effects of psychedelics and their therapeutic potential [251,254,256]. By addressing current challenges and fostering open dialogue about best practices, the field of psychedelic research holds significant promise for transforming ASD treatment paradigms and improving outcomes for affected individuals.

## 8. Conclusions

In conclusion, the review has provided a comprehensive overview of the potential use of psychedelics for managing Autism Spectrum Disorder. Existing pharmacological interventions for ASD are limited and do not directly target the core symptoms of the disorder. The emerging field of psychedelic science presents a new and exciting area of research, with preliminary findings showing promise in addressing the core symptoms of ASD. However, the review also demonstrates that research in this area is still in the early stages. There are still many unknowns regarding the therapeutic effects and safety of psychedelic medications, and the current findings need to be validated by further research. The safety of psychedelics is a primary concern, particularly for a vulnerable patient population such as individuals with ASD. Administration of psychedelics needs to be carried out under strict medical supervision and consideration for the potential risks and side effects of these medications. From an ethical perspective, the review has highlighted the various challenges and considerations associated with the use of psychedelics in the treatment of ASD, particularly in relation to obtaining informed consent, minimizing risks, and protecting the well-being of patients. Overall, it is crucial to identify and address the limitations of current research and to establish a solid foundation for future studies in this field. This includes adopting rigorous research methodologies, such as randomized controlled trials, and developing an integrative multi-disciplinary approach to investigate the therapeutic potentials of psychedelics for ASD. The future directions outlined in the review, such as investigating the neural mechanisms of psychedelic therapy and the potential for personalized medicine, will help inform researchers and policymakers in strategic planning for future ASD research. This will be essential in driving the field forward and in realizing the possibility of psychedelic-assisted therapies being translated from the laboratory to clinical practice. The review emphasized that the growing interest in psychedelic research for ASD necessitates an awareness of the ethical and regulatory challenges associated with potential clinical translation. This includes addressing the complex legal status of psychedelics, the need for clear and standardized regulations governing their research and clinical use, and the careful consideration of individual psychological and spiritual support in psychedelic therapies. The review has also showcased that the successful implementation of psychedelic-assisted therapies for ASD will require significant changes in clinical practice and in public attitudes towards the use of psychedelics. Education of health professionals and the public with up-to-date scientific evidence of the safety and efficacy of psychedelic medications will be an important steppingstone towards the acceptance and recognition of this novel treatment option for ASD. Finally, the review underscores the weight of responsibility in ensuring the ethical conduct of research in psychedelics by promoting transparency, community engagement, and the establishment of a supportive and safe research environment. Through an inclusive and ethical approach, the field of psychedelic science will be able to flourish and ultimately offer hope for new and effective treatments for ASD.

## Figures and Tables

**Table 1:** Psychedelics mentioned in the article with descriptions and chemical structures. This table provides an overview of the psychedelics mentioned in the article, including their descriptions and chemical structures. Each psychedelic is listed with a brief description of its properties and effects, along with an image of its chemical structure.

Psychedelic	Description	Structure
**LSD (Lysergic Acid Diethylamide)**	Known for its potent hallucinogenic effects, LSD alters perception, mood, and consciousness.	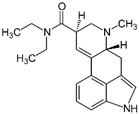
**Psilocybin**	Found in certain species of mushrooms, psilocybin is known for its introspective and mystical qualities.	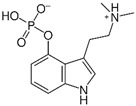
**DMT (Dimethyltryptamine)**	A naturally occurring psychedelic, DMT induces intense, short-lived visionary experiences.	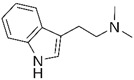
**MDMA (3,4-Methylenedioxymethamphetamine)**	Known for its empathogenic effects, MDMA enhances social interaction and emotional connection.	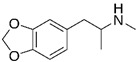
**Ibogaine**	Derived from the root bark of the African Tabernanthe iboga plant, ibogaine is being studied for its potential to treat addiction and promote neuroplasticity.	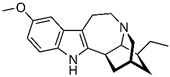
**Mescaline**	Found in the peyote cactus, mescaline induces profound alterations in perception and cognition.	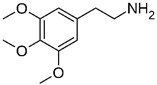
**Salvinorin A**	The active compound in Salvia divinorum, salvinorin A, is a potent kappa-opioid receptor agonist with dissociative effects.	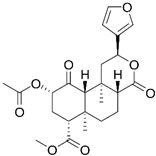
**2C-B (4-Bromo-2,5-dimethoxyphenethylamine)**	A synthetic psychedelic known for its effects on social behavior and anxiety.	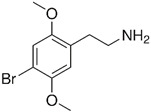
**Myristicin**	A compound found in nutmeg, myristicin has psychoactive properties and potential therapeutic effects.	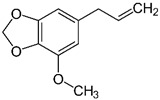

**Table 2:** Overview of widely used psychedelic compounds and mechanisms.

Compound	Primary Receptor Targets	Key Neurobiological Effect	Clinical Relevance
LSD	5-HT_2A_, 5-HT_1A_, D1, D2	Enhanced thalamocortical connectivity	Mood and perception modulation
Psilocybin	5-HT_2A_, 5-HT_1A_, 5-HT_2C_	Promotion of “pivotal mental states”	Treatment-resistant depression
MDMA	5-HT/DA release; Oxytocin	Increased social salience	PTSD; Social anxiety in ASD
DMT	5-HT_2A_, Sigma-1	Rapid synaptic remodeling	Short-acting neuroplasticity

**Table 3:** Potential therapeutic impact of psychedelics in ASD.

Symptom Domain	Proposed Mechanism	Potential Benefit in ASD
Social Communication	Oxytocin release; 5-HT modulation	Improved empathy and social interaction
Rigid Behaviors	Increased psychological flexibility	Reduction in rigid behavioral patterns
Sensory Processing	Thalamocortical circuit adjustment	Reduced sensory over-stimulation/anxiety
Neuroplasticity	BDNF upregulation; Dendritic growth	Long-term functional brain adaptation

**Table 4:** Positive and negative social effects of various psychedelics. This table summarizes the positive and negative social effects of various psychedelics mentioned in the article.

Psychedelic	Positive Social Effects	Negative Social Effects
LSD (Lysergic Acid Diethylamide)	Enhanced empathy, increased openness to social interactions, and improved social bonding	Potential for altered social perception, anxiety, and paranoia
Psilocybin	Enhanced emotional connection, increased empathy, improved social interaction, reduced social anxiety	Potential for anxiety, paranoia, and altered social perception
DMT (Dimethyltryptamine)	Enhanced empathy post-experience	Intense introspective experiences that may lead to social withdrawal during the experience
MDMA (3,4-Methylenedioxymethamphetamine)	Strongly enhances empathy and emotional connection, increases sociability, reduces social anxiety, promotes trust and closeness	Potential for emotional vulnerability, dependence, and post-use depression
Ibogaine	Improved social behavior through introspection and emotional processing, reduced social withdrawal	Intense introspective experiences that may be overwhelming, and potential for anxiety
Mescaline	Enhanced emotional connection, increased empathy, improved social interactions, and bonding	Potential for anxiety, paranoia, and altered social perception
Salvinorin A	Limited positive social effects due to its dissociative nature	Altered social perception, potential for anxiety and confusion
2C-B (4-Bromo-2,5-dimethoxyphenethylamine)	Enhanced sociability, increased empathy, improved emotional connection, reduced social anxiety	Potential for anxiety, paranoia, and altered social perception
Myristicin	Enhanced emotional connection and sociability	Risk of anxiety, paranoia, and confusion at high doses

## Data Availability

No new data were created or analyzed in this study.

## Abbreviations

The following abbreviations are used in this manuscript:

ABA	Applied Behavior Analysis
ADDM	Autism and Developmental Disabilities Monitoring
AMPA	α-amino-3-hydroxy-5-methyl-4-isoxazolepropionic acid
AND	Autism and Neurodevelopmental Disorders
ASD	Autism Spectrum Disorder
BDI	Beck Depression Inventory
BDNF	Brain-Derived Neurotrophic Factor
CDC	Centers for Disease Control and Prevention
CP	Cerebral Palsy
CSG	Clinical Science Group
DEA	Drug Enforcement Administration
DMT	N,N-Dimethyltryptamine
DSM	Diagnostic and Statistical Manual of Mental Disorders
DSM-5	Diagnostic and Statistical Manual of Mental Disorders, 5th Edition
EEG	Electroencephalography
ERK	Extracellular signal-regulated kinase
FDA	Food and Drug Administration
GAD-7	Generalized Anxiety Disorder 7-item scale
HCP	Healthcare Professional
HPPD	Hallucinogen Persisting Perception Disorder
HT	5-Hydroxytryptamine (serotonin)
IL	Interleukin
KOR	Kappa opioid receptor
LSD	Lysergic acid diethylamide
MDMA	3,4-Methylenedioxymethamphetamine
MDT	Mediodorsal nucleus of the thalamus
MMDA	3-Methoxy-4,5-methylenedioxyamphetamine
MRI	Magnetic Resonance Imaging
MRS	Magnetic Resonance Spectroscopy
NIMH	National Institute of Mental Health
PBS	Positive Behavior Support
PTSD	Post-Traumatic Stress Disorder
THC	Tetrahydrocannabinol
